# Editorial: Microbial therapeutics: harnessing the human microbiome for disease treatment and prevention

**DOI:** 10.3389/fmedt.2025.1751147

**Published:** 2025-12-12

**Authors:** Ali Asger Bhojiya, Abhinav Saurabh, Devendra Jain

**Affiliations:** 1 Faculty of Science, U.S. Ostwal P.G. College, Chittorgarh, India; 2 Clinical Research Center, Cardiovascular Branch/NHLBI, National Institutes of Health, Bethesda, MD, United States; 3 Department of Molecular Biology and Biotechnology, Rajasthan College of Agriculture, Maharana Pratap University of Agriculture and Technology, Udaipur, India

**Keywords:** cancer immunotherapy, disease prevention, fecal microbiota transplantation, gut-brain axis, human microbiome, microbial therapeutics, probiotics

## Introduction

Microbiome science has advanced dramatically during the last ten years, changing our knowledge of human health, disease causation, and precision medicine. Six scholarly contributions—five research reviews and one experimental study—that emphasize the diverse roles of the human microbiome in metabolic regulation, disease etiology, biomarker discovery, and the development of next-generation therapeutics are included under this research topic. When taken collectively, these papers highlight the quickly growing potential of therapies based on microbiomes across a variety of clinical specialties.

Ahmed et al. provide an exhaustive evaluation of microbiome-centric therapies for metabolic disorders in the first article of this research area. They discuss the growth in obesity, type 2 diabetes, non-alcoholic fatty liver disease, and metabolic syndrome worldwide. The authors describe how metabolic dysfunction is caused by gut dysbiosis, which is characterized by decreased diversity, disturbed synthesis of short-chain fatty acids, increased intestinal permeability, and persistent low-grade inflammation. Probiotics, prebiotics, synbiotics, postbiotics, engineered microbial consortia, and fecal and vaginal microbiota transplantation are among the novel treatment strategies that are summarized in this review. The authors highlight enduring issues such as interindividual variability, strain specificity, dosage optimization, regulatory monitoring, and the requirement for individualized, multi-omics-integrated therapy regimens despite encouraging pre-clinical and early clinical results.

Bautista et al. provide a comprehensive examination of the human microbiome in clinical translation, from laboratory to clinical application, building on the translational potential of microbiome science. The authors emphasize how microbiome profiling is changing immunological homeostasis, cardiometabolic health, neuropsychiatric disorders, early-life development, and cancer therapeutic responsiveness by utilizing developments in multi-omics, computational modeling, and experimental biology. While addressing important obstacles like high interindividual variability, poor functional annotation, and a lack of validated biomarkers, they also covered innovative treatment approaches, including phage therapy, live biotherapeutics, precision nutrition, and microbiota transplantation. The study emphasizes that in order to fully realize the clinical promise of microbiome-driven therapies, consistent techniques, regulatory harmonization, and long-term cohort studies are required.

Upadhyay et al. investigate the optimization of *Lactobacillus rhamnosus* CW40's bacteriocin production and assess its antibacterial and therapeutic potential in a study centered on microbial biotherapeutics. The researchers found that strain CW40, which produced an 8 kDa protein with significant antibacterial efficacy against *Bacillus subtilis, Bacillus cereus*, and *Escherichia coli*, was a powerful bacteriocin producer after screening 47 LAB isolates. At 37°C and pH 7, bacteriocin production was at its peak, with an activity of 4,098 AU/ml. Its proteinaceous composition was confirmed by enzyme sensitivity tests, and the strain showed broad antibiotic resistance and bile salt tolerance. This study emphasizes the potential of bacteriocins derived from CW40 as natural food biopreservatives and therapeutic agents against antibiotic-resistant illnesses and foodborne pathogens.

Dakal et al. offer a technological and computational viewpoint by reviewing machine-learning techniques, artificial intelligence, and sophisticated computer tools for gut microbiota and biomarker detection. The authors explain how integrated multi-omics (metagenomics, metabolomics, and metaproteomics) in conjunction with deep learning and machine learning models can improve therapy prediction accuracy, uncover novel disease biomarkers, and decipher intricate microbial interaction networks. In order to facilitate the development of individualized and precise medical interventions, they highlight the significance that AI-driven models play in predicting individual reactions to medications that target the microbiome.

Alam et al. examine microbiome-based treatments for Parkinson's disease (PD) in light of the role that microbiome dysbiosis plays in neurodegeneration. The authors talk about how PD incidence and progression are influenced by disruptions in the gut-brain axis, including increased intestinal permeability, chronic inflammation, oxidative stress, neurotransmitter imbalance, and α-synuclein aggregation. They draw attention to inflammatory mediators, including TNF-α, IL-1β, and IL-6, microbial taxa linked to Parkinson's disease, and the impact of the microbiome on the metabolism of dopamine and levodopa. Alongside nutrition-based therapies like Mediterranean and ketogenic diets, therapeutic approaches including probiotics, prebiotics, synbiotics, postbiotics, and fecal microbiota transplantation are being investigated.

Lastly, a thorough scoping review of third-millennium gut microbiome studies on celiac disease is provided by Luz and Pereira. The authors point out significant variation in sample types, microbiota identification techniques, and reporting standards after examining 48 studies that were published between 2000 and 2023. Even though some patterns, like changed Pseudomonadota abundance, seem to be consistent, inconsistent research makes it difficult to agree on the microbiological markers of celiac disease. To improve repeatability and make it possible to make more accurate predictions about health consequences, the review recommends standardizing sampling techniques, sequencing protocols, bioinformatics workflows, and metadata reporting.

In conclusion, microbial therapeutics represent a transformative paradigm in medicine, offering novel opportunities for disease prevention, treatment, and health optimization. While significant scientific and regulatory challenges persist, the ongoing convergence of microbiome science and clinical practice holds the promise of reshaping contemporary healthcare. Future research must prioritize mechanistic studies, controlled clinical trials, and the development of precision microbiome interventions tailored to individual microbial signatures and disease phenotypes. As this field matures, microbial therapeutics are poised to become an integral component of personalized medicine and public health strategy.

